# High quality draft genomes of the *Mycoplasma mycoides* subsp. *mycoides* challenge strains Afadé and B237

**DOI:** 10.1186/s40793-015-0067-0

**Published:** 2015-10-29

**Authors:** Anne Fischer, Ivette Santana-Cruz, Jan Hegerman, Hadrien Gourlé, Elise Schieck, Mathieu Lambert, Suvarna Nadendla, Hezron Wesonga, Rachel A. Miller, Sanjay Vashee, Johann Weber, Jochen Meens, Joachim Frey, Joerg Jores

**Affiliations:** International Livestock Research Institute, PO Box 30709, 00100 Nairobi, Kenya; International Centre for Insect Physiology and Ecology, PO Box 30772, 00100 Nairobi, Kenya; Institute for Genome Sciences, University of Maryland School of Medicine, 801 W. Baltimore Street BioPark II, 21201 Baltimore, MD USA; Institute of Functional and Applied Anatomy, Hannover Medical School, Hannover, Germany; Biomedical Research in Endstage and Obstructive Lung Disease Hannover (BREATH), Member of the German Center for Lung Research (DZL), Hannover, Germany; REBIRTH Cluster of Excellence, Hannover, Germany; Department of Animal Breeding and Genetics, SLU Global Bioinformatics Centre, Swedish University of Agricultural Sciences, SE75007 Uppsala, Sweden; Kenya Agricultural and Livestock Research Organization (KALRO) Muguga, PO Box 32-00902, Kikuyu, Kenya; Department of Food Science, Cornell University, Ithaca, NY USA; J. Craig Venter Institute, 9704 Medical Center Drive, 20850 Rockville, MD USA; Lausanne Genomic Technologies Facility Center for Integrative Genomics, University of Lausanne, 1015 Lausanne, Switzerland; Institute for Microbiology, Department of Infectious Diseases, University of Veterinary Medicine Hannover, Hannover, Germany; Institute of Veterinary Bacteriology, University of Bern, CH-3001 Bern, Switzerland

**Keywords:** *Mycoplasma mycoides* subsp. *mycoides*, Challenge strain, Genome, Contagious bovine pleuropneumonia, Protrusion

## Abstract

Members of the *Mycoplasma mycoides* cluster’ represent important livestock pathogens worldwide. *Mycoplasma mycoides * subsp. *mycoides* is the etiologic agent of contagious bovine pleuropneumonia (CBPP), which is still endemic in many parts of Africa. We report the genome sequences and annotation of two frequently used challenge strains of *Mycoplasma mycoides* subsp. *mycoides,* Afadé and B237. The information provided will enable downstream ‘omics’ applications such as proteomics, transcriptomics and reverse vaccinology approaches. Despite the absence of *Mycoplasma pneumoniae* like cyto-adhesion encoding genes, the two strains showed the presence of protrusions. This phenotype is likely encoded by another set of genes.

## Introduction

The ‘*Mycoplasma mycoides* cluster’ comprises five species/subspecies, *Mycoplasma mycoides* subsp. *mycoides*, *Mycoplasma leachii*, *Mycoplasma mycoides* subsp. *capri*, *Mycoplasma capricolum* subsp. *capripneumoniae* and *Mycoplasma capricolum* subsp. *capricolum* [1, 2]. Among them, *Mycoplasma mycoides* subsp. *mycoides*, the causative agent of contagious bovine pleuropneumonia (CBPP), is an economically very important bacterial bovine pathogen in sub-Saharan Africa. CBPP was first described in Europe already in 1773 [3], and the causative *Mycoplasma* was then cultivated and characterized in 1898 in Europe [4]. It has been shown that it spread from Europe to North America, Africa, Australia and Asia via livestock movements. Currently the disease is endemic and widespread in sub-Saharan Africa, ranging from western, central to eastern Africa. In Europe the last outbreaks were reported in Spain, Italy, Portugal and France in the 1980s and 1990s [5]. In comparison to other members of the *‘﻿ Mycoplasma mycoides* cluster’, with the exception of *Mycoplasma capricolum* subsp. *capripneumoniae*, *Mycoplasma mycoides* subsp. *mycoides* shows limited sequence diversity, probably due to its recent emergence about 300 years ago [5, 6].

Currently the complete genomes of only three *Mycoplasma mycoides* subsp*. mycoides* strains have been deposited in GenBank, the type strain PG1 [7], which is often used in laboratories but which is considered to be avirulent, the Australian outbreak strain Gladysdale [8] and a European outbreak strain 57/13 [9]. PG1 has been shown to differ genetically and phenotypically from field stains of *Mycoplasma mycoides* subsp*. mycoides*, showing attenuated cytotoxicity and reduced adhesion to bovine epithelial cells [5, 10, 11], most likely because of the multiple *in vitro* passages this strain underwent before being deposited in the strain collections. In particular strain PG1 contains 2 large 24 kb repeats while 27 field strains isolated from three different continents only contain one [11]. Strain Gladysdale was isolated from Australia around 1953 [12]. Strain 57/13 was isolated in Italy in 1992. Neither of these three strains, therefore, represent virulent African strains. The genetic diversity of *Mycoplasma mycoides* subsp*. mycoides* strains has been reported to be highest in Africa [5] where the disease is present in many countries of sub-Saharan Africa [13]. We sequenced and annotated the genomes of two virulent African strains Afadé and B237, which are frequently used as challenge strains in animal experiments [14–18]. The strains have been re-isolated directly from experimentally infected animals and have not been exposed to subsequent passaging beyond filter-cloning to promote uniformity before genomic DNA was isolated for sequencing. The genomic sequence information from this work will contribute to comparative genomic analyses and therefore the characterization of the core and pan genome of the ‘*Mycoplasma mycoides* cluster’ and *Mycoplasma mycoides* subsp*. mycoides* in particular. The genomic information will also be useful for downstream ‘omics’ applications, such as proteomics, transcriptomics and reverse vaccinology approaches.

## Organism information

### Classification and features

**Fig. 1:**
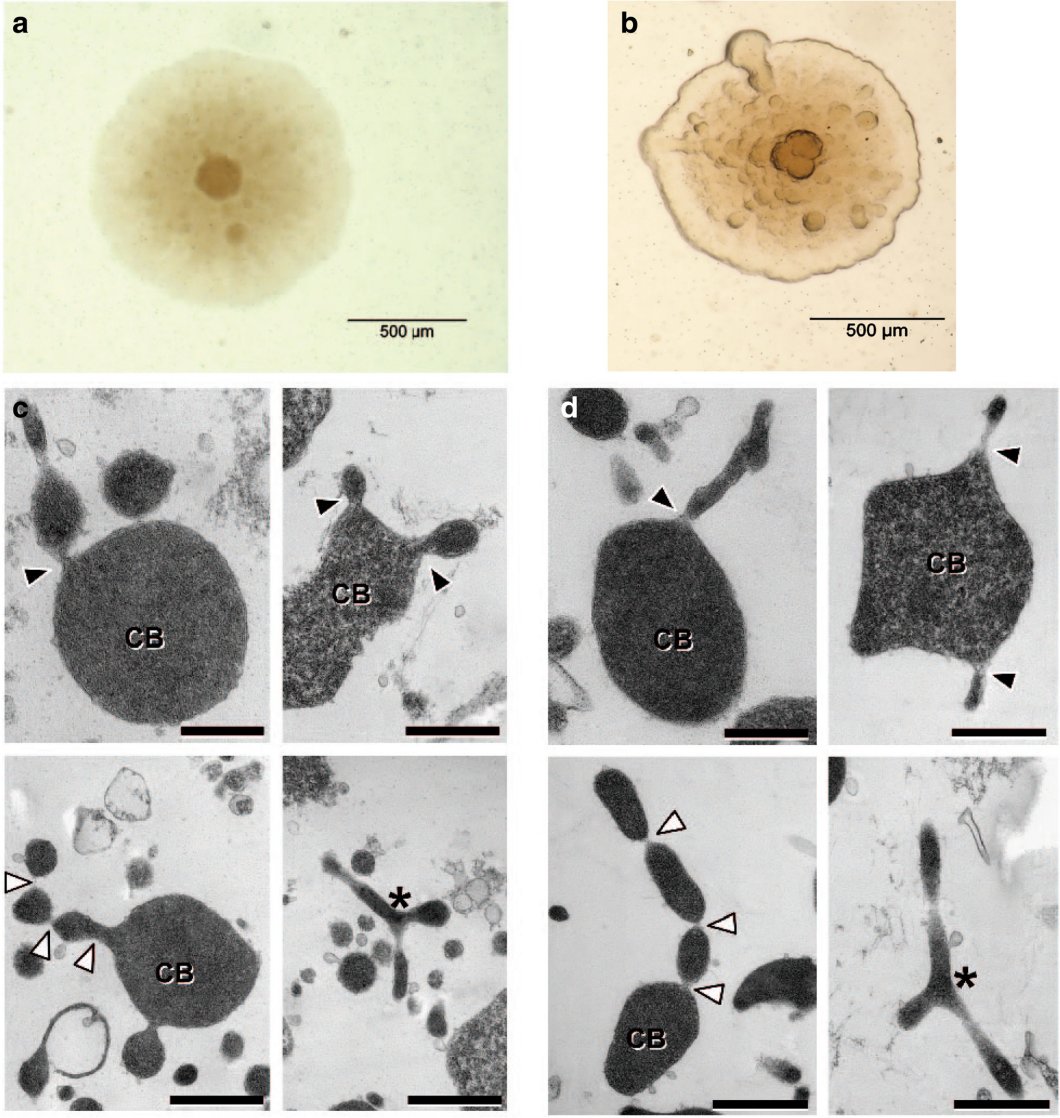
(quarter page, single column): Typical fried egg-shaped colony of *Mycoplasma.* **a** Afadé, **b** B237. Transmission electron microscopy of Afadé (**c**) and B237 (d). Ultrathin sections reveal cell bodies (CB) and thin protrusions (*black arrowheads*, *top left*). Multiple protrusions can originate from one cell body (*top right*). Multiple constrictions along protrusions lead to a necklace-like appearance in some regions (*bottom left*, *white arrowheads*). Branching along the protrusions occurs (*bottom* *right, asterisk*)

*Mycoplasma mycoides* subsp*. mycoides* is an obligate parasite, which resides in the respiratory tract of animals. It is a non-motile, non-sporulating bacterium. It lacks a cell wall and has a pleomorphic shape. Transmission electron microscopy images were generated for both Afadé and B237 strains (Fig. 1). Cell pellets were fixed in 150 mM HEPES, pH 7.35, containing 1.5 % formaldehyde and 1.5 % glutaraldehyde for 30 min at RT and at 4 ° over night. After dehydration in acetone and embedding in EPON, ultrathin sections of 40 nm were mounted on formvar-coated coppergrids, poststained with uranyl acetate and lead citrate [19] and observed in a Morgagni TEM (FEI). Images were taken with a side mounted Veleta CCD camera.

Interestingly the transmission electron microscopy revealed protrusions resembling the attachment organelle observed in *Mycoplasma**pneumonia* [20–23]. The physiological function of these protrusions and branching phenotype needs to be defined in future studies. The general features of *Mycoplasma mycoides* subsp*. mycoides* strains Afadé and B237 are presented in Table 1 and Appendix: Table 6.

**Table 1:** Classification and general features of *Mycoplasma mycoides subsp. mycoides* strains Afadé and B237

MIGS ID	Property	Term	Evidence code^a^
	Classification	Domain *Bacteria*	TAS [39]
		Phylum *Firmicutes*	TAS [40]
		Class *Tenericutes*	TAS [41–44]
		Order *Mycoplasmatales*	TAS [45, 46]
		Family *Mycoplasmataceae*	TAS [46]
		Genus *Mycoplasma*	IDA
		Species *Mycoplasma mycoides*	IDA [4]
		Subspecies *Mycoplasma mycoides* subsp*. mycoides*	IDA [4]
		Strains Afadé and B237	
	Cell shape	Pleomorph	IDA
	Motility	Nonmotile	IDA
	Sporulation	Nonspore-forming	IDA
	Temperature range	30–42 °C	IDA
	Optimum temperature	38.5 °C	IDA
	pH range; optimum	6.5 – 8.5; 7.5	IDA
	Carbon Source	Not determined since strains require complex media including serum for growth	-
	Energy Source	Not determined since strains require complex media including serum for growth	-
MIGS-6	Habitat	Respiratory tract	IDA
MIGS-6.3	Salinity	0.09 %, no growth was obtained at salinities ≥0.5 M NaCl	IDA
MIGS-22	Oxygen Requirement	Facultative anaerobe	[42]
MIGS-15	Biotic relationship	Pathogen	-
MIGS-14	Pathogenicity	Etiological agent of Contagious Bovine Pleuropneumonia (CBPP)	-
MIGS-4	Geographic location	Cameroon (Afadé), Kenya (B237)	[3]
MIGS-5	Sample collection time	1965 (Afadé), 1997 (B237)	-
MIGS-4.1	Latitude	Northern Cameroon (Afadé) 01°03′S (B237)	
MIGS-4.2	Longitude	N/A (Afadé) 37°05′E (B237)	
MIGS-4.3	Depth	N/A	
MIGS-4.4	Altitude	N/A (Afadé), 1631 m (B237)	

^a^Evidence codes - IDA: Inferred from Direct Assay; TAS: Traceable Author Statement (i.e., a direct report exists in the literature); NAS: Non-traceable Author Statement (i.e., not directly observed for the living, isolated sample, but based on a generally accepted property for the species, or anecdotal evidence). These evidence codes are from the Gene Ontology project [47]

We previously confirmed that both strains Afadé and B237 are *Mycoplasma mycoides* subsp*. mycoides* using phenotypic growth characteristics, species-specific PCR and a Multi-Locus Sequence Typing (MLST) method [5, 6]. *Mycoplasma mycoides* subsp*. mycoides* strain Afadé originates from Northern Cameroon and was isolated at the Farcha laboratories in Tchad in 1965 [24]. It has since served for several experimental infections [14–18]. The filter-cloned strains used for this sequence analysis were re-isolated from experimentally infected cattle [14, 17] that showed severe clinical signs and pathomorphologic lesions typical of CBPP. *Mycoplasma mycoides* subsp*. mycoides* strain B237 was originally isolated in 1997 in Thika, Kenya, by the Kenya Agricultural Research Institute (KARI).

**Fig. 2:**
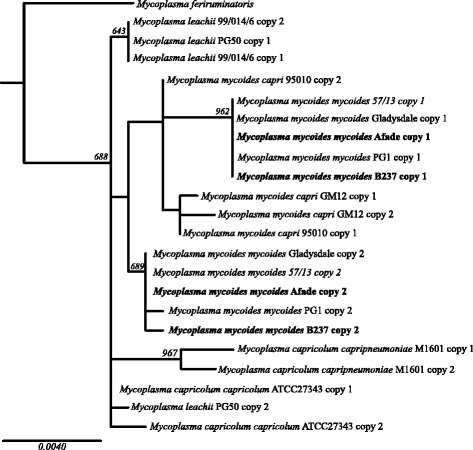
(half page, 2 columns): Phylogenetic tree based on 16S rRNA sequences showing the relationship between *Mycoplasma mycoides* subsp*. mycoides* strains Afadé and B237 with members of the ‘*Mycoplasma mycoides* cluster’ and their closest relatives. The alignment length was 1,439 bp. The tree was generated with PhyML v.3.0 [48] using the HKY85 model of evolution and with 1,000 bootstrap values. Only boostrap values over 500 are shown.

Figure 2 shows a phylogenetic tree of the 16S rRNA sequences. 16S rRNA gene sequences from *Mycoplasma mycoides* subsp*. mycoides* strains Gladysdale, 57/13 and PG1, *Mycoplasma mycoides capri* strains 95010 and GM12, *Mycoplasma capricolum* subsp. *capricolum* strain ATCC27343, *Mycoplasma capricolum* subsp*. capripneumoniae* strain M1601, *Mycoplasma leachii* strains 99/014/6 and PG50, *Mycoplasma**feriruminatoris* strain G5847 (Accession numbers: CP002107, CP010267, NC_005364, NC_015431, NZ_CP001668, NC_007633, CM001150, NC_017521, ANFU01000033, NC_014751, respectively) were retrieved from GenBank. All *Mycoplasma* genome sequences retrieved from GenBank have two copies of 16S rRNA each, with the exception of *Mycoplasma**feriruminatoris*, where two copies are present but are not resolved in the draft genome [25].

## Genome sequencing information

### Genome project history

The sequencing and quality assurance was performed at Lausanne Genomic Technologies Facility, Center for Integrative Genomics, University of Lausanne, Switzerland. The assemblies and finishing were done at the Institute for Genome Sciences and International Livestock Research Institute. Functional annotation was produced by the Institute for Genome Sciences Analysis Engine [26] (http://www.igs.umaryland.edu/research/bioinformatics/analysis/index.php). Table 2 presents the project information and its association with MIGS version 2.0 compliance [27].

**Table 2:** Project information

MIGS ID	Property	Term	Term
MIGS-31	Finishing quality	High-quality draft	High-quality draft
MIGS-28	Libraries used	1. Illumina Paired End 7,078,010 reads; Average read length 295 bp; Average insert size 725 bp.	1. PacBio 59,775 reads; Average read length 2674 bp
		2. PacBio 65,280 reads, 2853 bp average read length;	
MIGS-29	Sequencing platforms	Illumina MiSeq, Pacific Biosciences R.S.	Illumina MiSeq, Pacific Biosciences R.S.
MIGS-31.2	Fold coverage	24X	23X
MIGS-30	Assemblers	Celera Assembler v.7	Celera Assembler v.7
MIGS-32	Gene calling method	Prodigal	Prodigal
	Genbank ID	LAEX00000000	LAEW00000000
	Date of Release	20-Mar-15	20-Mar-15
	BIOPROJECT	PRJNA272775	PRJNA272471
MIGS 13	Source Material Identifier	ILRI_Azizi_biobank Strain Afadé	ILRI_Azizi_biobank Strain B237
	Project relevance	Challenge strains of CBPP	Challenge strains of CBPP

### Growth conditions and genomic DNA preparation

Both strains were grown in PPLO medium (Difco, Cat no. 255420) supplemented with 20 % heat-inactivated horse serum (Sigma, Cat. No. H1138), 0.5 % glucose, 0.03 % penicillin G, 20 mg/ml thallium acetate and 0.9 g/L yeast extract at 37 °C.

Liquid cultures of *Mycoplasma* were filter cloned using a 0.22 μm filter to disrupt possible cell aggregates. A serial dilution (1/10 - 1/10,000,000,000) was made immediately and 50 μl was plated on PPLO agar.

After 3–4 days of incubation at 37 °C, a single colony was picked and was used to inoculate 4 ml of PPLO medium which was aliquoted and stored at −80 °C.

Filter cloned *Mycoplasma* were grown overnight in 100 ml PPLO medium at 37 °C. Before entering the stationary growth phase the culture was centrifuged at 2,862 g for 1 h, and the pellet was resuspended in 2.5 ml of TNE buffer (0.01 M Tris–HCl, pH 8.0; 0.01 M NaCl; 0.01 M EDTA). Subsequently 50 μl SDS (10 %) and 50 μl Proteinase K (20 mg/ml) were added and the tubes were incubated at 37 °C for 2 h. After addition of 26 μl of 100 mM PMSF the tubes were incubated 15 min at room temperature, 25 μl of RNase A (10 mg/ml) was added, followed by incubation at 37 °C for 1 hr. Sodium acetate and Phenol Saturated Buffer was added (25 μl of NaOAc 1.5 M pH 5.2, and 2250 μl of Phenol), the solution was mixed by vortexing and centrifuged at 15,870 g for 10 min. The top phase was transferred to a new tube and mixed with Phenol:Chloroform:Isoamyl Alcohol Buffer (Phenol:Chloroform:Isoamyl Alcohol; 25:24:1) followed by another centrifugation at 15,870 g for 10 min and again the top phase was transferred to a new tube. Finally, the DNA was precipitated with isopropanol, washed with 70 % ethanol, dried and resuspended in 200 μl of 2 mM Tris, 0.2 mM EDTA.

### Genome sequencing and assembly

The genome sequence of *Mycoplasma mycoides* subsp*. mycoides* strain Afadé was generated using a combination of Pacific Biosciences R.S. (PacBio) sequencing (65,280 reads/2853 bp average read length) and Illumina MiSeq sequencing (7,078,010 reads/295 average read length) down-sampled to cover 50 times the expected genome size. The sequencing errors of the long PacBio single-molecule reads were corrected with the shorter, high accuracy Illumina reads using the Celera Assembler (CA) pacbio correction module PBcR (version 7.0, [28]). The resulting corrected PacBio reads were randomly sampled to 25 genome fold and assembled using CA (version 7.0, [29]) and yielded 18 contigs with a total size of 1,278,455 bp. Eight contigs comprised the draft genome of strain Afadé.

The whole genome sequence of *Mycoplasma mycoides* subsp*. mycoides* strain B237 was obtained using PacBio sequencing (59,775 reads/2674 average read length). Pacbio reads were corrected with PBcR self-correction module. Corrected reads randomly sampled to 25 genome fold were assembled with CA and yielded 2 contigs with total size of 1,208,895 bp. One long contigs comprises the entire genome and contained the other contig (5091 bp) in a repeat region. The final genome sequences had a 24-fold coverage for Afadé and 23-fold coverage for B237.

The contigs of both assemblies were aligned against the two *Mycoplasma mycoides* subsp*. mycoides* reference genomes of Gladysdale [8] and PG1 [7] available in Genbank (CP002107, NC_005364) using mummer [30] and we noticed that all small contigs (<15,000 bp) aligned to places already covered in other bigger contigs. On closer inspection, most of these contigs aligned to a previously characterized 26 kb region [11], consisting of a tandem repeat of three 8 kb segments, interspersed with transposon elements. Due to its repetitive nature, this 26 kb region was not clearly resolved during the assembly process. In order to resolve part of it, we were able to design unique primer pairs and amplify two long-range PCRs fragments of 4,800 and 5,200 bp respectively. For each genome, both Sanger derived sequences were aligned to the assembled genomes before and after polishing with multiple iterations of the PacBio Quiver algorithm (version 0.9.0 [31]). We verified that in the regions covered by the Sanger sequences, all substitution mismatches were resolved by Quiver, however we manually fixed a few indels present in the post polishing alignment, which were not corrected by Quiver.

### Genome annotation

Open reading frames (ORFs) were predicted using Prodigal 2.50 [32]. Functional annotation was produced by the Institute for Genome Sciences Analysis Engine [26].

We annotated the small contigs overlapping bigger ones described above separately and noticed that these contigs had more ambiguous characters and ORFs that were on average half the size of the corresponding ORFs in larger contigs (498 nt versus 920 nt). This was due to insertions and deletions. We therefore excluded the small contigs from the assemblies and report 1 contig for *Mycoplasma mycoides* subsp*. mycoides* strain B237 and 8 contigs for *Mycoplasma mycoides subsp. mycoides* strain Afadé.

We also reannotated the genomes of *Mycoplasma mycoides* subsp*. mycoides* strain PG1, *Mycoplasma mycoides* subsp*. mycoides* strain Gladysdale and *Mycoplasma mycoides* subsp. *mycoides* strain 57/13 using the same Engine, for ease of comparison.

## Genome properties

The genomes of *Mycoplasma mycoides* subsp*. mycoides* strain Afadé and B237 have a total size of 1,190,241 bp and 1,203,804 bp, respectively. The GC-content of both genomes is 23.9 %. Both strains have two copies of the 12 kb and 13 kb repeat described in [11], the difference in size between the two genomes is therefore not due to a missing copy in Afadé.

A total of 1,124 ORFs as well as 30 tRNA and 2 copies of the 23S, 16S and 5S rRNA operons were predicted. The average gene length is 920 bp and 927 bp for Afadé and B237, respectively. The coding density of the genome is 86.7 %. Signal peptides were detected using pSortb v3.0 [33] and LipoP v1.0 [34]. Transmembrane helices were detected with the TMHMM server v2.0 [35, 36]. CRISPR repeats were searched with the CRISPR Finding program online. The properties and the statistics of both genomes are summarized in Tables 3, 4, 5.

**Table 3:** Summary of the B237 and Afadé genomes: one circular chromosome

Strain	Size (Mb)	Topology	INSDC identifier
Afadé	1,190,241	8 contigs	LAEX00000000
B237	1,203,804	Circular	LAEW00000000

**Table 4:** Nucleotide content and gene count levels of the genome

Strain	Afadé		B237	
Attribute	Value	% of total^a^	Value	% of total^a^
Genome Size (bp)	1,190,241	100.00	1,203,804	100.00
DNA coding (bp)	1,032,189	86.70	1,043,698	86.70
DNA G + C (bp)	284,536	23.90	287,709	23.90
DNA scaffolds	na	na	na	na
Total genes	1156	100.00	1157	100.00
Protein-coding genes	1120	96.89	1121	96.89
rRNA genes	6	5.19	6	5.19
Pseudogenes	0	0	0	0
Genes in internal clusters	na	na	na	na
Genes with function prediction	687	59.43	693	59.90
Genes assigned to COGs	681	58.71	693	59.9
Genes with Pfam domains	389	33.65	355	30.68
Genes with signal peptides	74	6.40	74	6.40
Genes with transmembrane helices	234	20.24	241	20.83
CRISPR repeats	0.00	0.00	0.00	0.00

^a^The total is based on either the size of the genome in base pairs or the total number of protein coding genes in the annotated genome

**Table 5:** Number of genes associated with the 25 general COG functional categories

Code	Value	% of total^a^	Value	% of total^a^	Description
Strain	Afadé		B237		
J	141	12.19	139	12.01	Translation, ribosomal structure and biogenesis
A	0	0.00	0	0.00	RNA processing and modification
K	34	2.94	34	2.94	Transcription
L	50	4.32	50	4.32	Replication, recombination and repair
B	0	0.00	0	0.00	Chromatin structure and dynamics
D	9	0.78	8	0.69	Cell cycle control, Cell division, chromosome partitioning
Y	0	0.00	0	0.00	Nuclear structure
V	12	1.04	13	1.12	Defense mechanisms
T	15	1.30	15	1.30	Signal transduction mechanisms
M	27	2.34	33	2.85	Cell wall/membrane biogenesis
N	8	0.69	9	0.78	Cell motility
Z	0	0.00	0	0.00	Cytoskeleton
W	0	0.00	0	0.00	Extracellular structures
U	5	0.43	6	0.52	Intracellular trafficking and secretion
O	26	2.25	25	2.16	Posttranslational modification, protein turnover, chaperones
C	29	2.51	28	2.42	Energy production and conversion
G	71	6.14	70	6.05	Carbohydrate transport and metabolism
E	44	3.81	42	3.63	Amino acid transport and metabolism
F	32	2.77	32	2.77	Nucleotide transport and metabolism
H	30	2.60	29	2.51	Coenzyme transport and metabolism
I	14	1.21	14	1.21	Lipid transport and metabolism
P	39	3.37	48	4.15	Inorganic ion transport and metabolism
Q	1	0.09	1	0.09	Secondary metabolites biosynthesis, transport and catabolism
R	45	3.89	45	3.89	General function prediction only
S	6	0.52	6	0.52	Function unknown
-	101	8.74	105	9.08	Other COG categories
-	442	38.24	431	37.25	Not in COGs

^a^The total is based on the total number of protein coding genes in the annotated genome

## Insights from the genome sequence

**Fig. 3:**
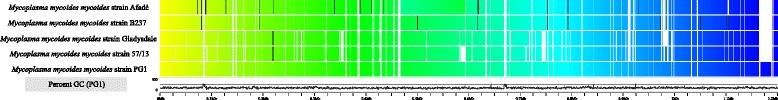
(quarter page, two columns): Synteny gradient display for the four available *Mycoplasma mycoides* subsp*. mycoides* genomes, using PG1 as a reference. A white bar in the reference denotes a region with no gene annotation. The matching genes are colored based on the relative position in their respective genomes (*yellow* for the beginning and *blue* for the end). Genes shown in black are part of a paralogous cluster in their respective genome and therefore do not have a single native location. The GC-content in % is plotted for the reference genome

The genomes of the two African strains *Mycoplasma mycoides* subsp*. mycoides* Afadé and B237 were compared to the three previously sequenced *Mycoplasma mycoides* subsp*. mycoides* strains Gladysdale, PG1 and 57/13 using CloVR and Sybil [37, 38]. Figure 3 shows a synteny gradient of the aligned genomes. Although there are a high number of transposable elements in all genomes, no major rearrangements have been observed. These results fit well with the very recent emergence of the pathogen, estimated to be as young as 300 years, and the narrow host specificity of *Mycoplasma mycoides* subsp*. mycoides* [5].

The core genome length is 1,148,950 bp. A total of 773 SNPs were identified when comparing the five core genomes. Only 72 SNPs distinguish B237 from Afadé. Two hundred and sixty six SNPs separate the Australian and European strains Gladysdale and 57/13. PG1 is the most distant from the other four genomes with 399, 483, 465 to 425 SNPs when compared to Afadé, Gladysdale, 57/13 and B237, respectively. This confirms previous reports [5].

We looked for homologs to the Cytadhesin proteins P1, P30, P40. P65, P90, HMW1 and HMW3 from *Mycoplasma pneumoniae* in the Afadé and B237 proteomes using blastp. No significant hits were found for any of the proteins. Other proteins might be involved in the adhesion process and will need to be identified and characterized.

## Conclusions

The genomes of the two African strains as expected differ from the laboratory type strain PG1, the European outbreak strain 57/13 and the Australian outbreak strain Gladysdale. Therefore these genome sequences should be included in subsequent genome comparisons and ‘omics’ studies. The presence of protrusions and branching phenotypes in these two Mycoplasmas but the absence of protein encoding genes similar to the ones characterized in *Mycoplasma pneumoniae* indicates that other/novel proteins in the *Mycoplasma* genomes encode the development of protrusions and branching.

## Appendix

**Table 6:** Associated MIGS record

MIGS-ID	field name	description	description
Strain		Afadé	B237
MIGS-1	Submit to INSDC/Trace archives	LAEX00000000	LAEW00000000
1.1	PID	PRJNA272471	PRJNA272775
1.2	Trace Archive		
MIGS-2	MIGS CHECK LIST TYPE		
MIGS-3	Project Name	High quality draft genomes of the *Mycoplasma mycoides* subsp*. mycoides* challenge strains Afadé and B237	High quality draft genomes of the *Mycoplasma mycoides* subsp*. mycoides* challenge strains Afadé and B237
MIGS-4	Geographic Location	Cameroon	Kenya
4.1	Latitude	not reported	01°03′S
4.2	Longitude	not reported	37°05′E
4.3	Depth	na	na
4.4	Altitude	not reported	1631 m
MIGS-5	Time of Sample collection	not reported	not reported
MIGS-6	Habitat (EnvO)	Respiratory tract	Respiratory tract
6.1	temperature	38.5	38.5
6.2	pH	6.5–8.5	6.5–8.5
6.3	salinity	0.09 %	0.09 %
6.4	chlorophyll	na	na
6.5	conductivity	na	na
6.6	light intensity	na	na
6.7	dissolved organic carbon (DOC)	na	na
6.8	current	na	na
6.9	atmospheric data	na	na
6.1	density	na	na
6.11	alkalinity	na	na
6.12	dissolved oxygen	na	na
6.13	particulate organic carbon (POC)	na	na
6.14	phosphate	na	na
6.15	nitrate	na	na
6.16	sulfates	na	na
6.17	sulfides	na	na
6.18	primary production	na	na
MIGS-7	Subspecific genetic lineage	strain	strain
MIGS-9	Number of replicons	1	1
MIGS-10	Extrachromosomal elements	none	none
MIGS-11	Estimated Size	1.2 MB	1.2 Mb
MIGS-12	Reference for biomaterial or Genome report	primary genome report	primary genome report
MIGS-13	Source material identifiers		
MIGS-14	Known Pathogenicity	Contagious Bovine Pleuropneumonia	Contagious Bovine Pleuropneumonia
MIGS-15	Biotic Relationship	obligate parasite	obligate parasite
MIGS-16	Specific Host	Cattle	Cattle
MIGS-17	Host specificity or range (taxid)	9903	9903
MIGS-18	Health status of Host	Sick	Sick
MIGS-19	Trophic Level	heterotroph	heterotroph
MIGS-22	Relationship to Oxygen	anaerobic	anaerobic
MIGS-23	Isolation and Growth conditions	*optional: reference may be provided if applicable*	*optional: reference may be provided if applicable*
MIGS-27	Nucleic acid preparation		
MIGS-28	Library construction		
28.1	Library size		
28.2	Number of reads		
28.3	vector		
MIGS-29	Sequencing method	Illumina Miseq 300PE and PacBio	PacBio
MIGS-30	Assembly		
30.1	Assembly method	Celera assembler v7.0	Celera assembler v7.0
30.2	estimated error rate		
30.3	method of calculation		
MIGS-31	Finishing strategy	High-quality draft	High-quality draft
31.1	Status		
31.2	coverage	25x	25x
31.3	contigs	7	1
MIGS-32	Relevant SOPs		
MIGS-33	Relevant e-resources		

## Abbreviations

CBPP: Contagious bovine pleuropneumonia

## References

[1] Manso-Silvan L, Vilei EM, Sachse K, Djordjevic SP, Thiaucourt F, Frey J (2009). *Mycoplasma leachii* sp. nov. as a new species designation for *Mycoplasma* sp. bovine group 7 of Leach, and reclassification of *Mycoplasma mycoides* subsp. *mycoides* LC as a serovar of *Mycoplasma mycoides* subsp. *capri*. Int J Syst Evol Microbiol.

[2] Krieg NR, Ludwig W, Whitman WB, Hedlund BP, Paster BJ, Staley JT (2010). Bergey's manual of systematic bacteriology. Volume 4.

[3] de Haller A. De Lue Bovilla Agri Bernensis Commentatio. Novi commentarii Societatis Regiae Scientiarum Gottingensis. Goettingen State University, Goettingen, Germany 1773:25–43.

[4] Hutyra F, Marek J, Manninger R. Diseases of Domestic Animals. Contagious Bovine Pleuropneumonia. Greig JR, Mohler JR, Eichhorn A, editors. London: Balliere, Tindal and Cox; 1938.

[5] Dupuy V, Manso-Silvan L, Barbe V, Thebault P, Dordet-Frisoni E, Citti C (2012). Evolutionary history of contagious bovine pleuropneumonia using next generation sequencing of *Mycoplasma mycoides* subsp. *mycoides* “Small Colony”. PLoS One.

[6] Fischer A, Shapiro B, Muriuki C, Heller M, Schnee C, Bongcam-Rudloff E (2012). The origin of the ‘*Mycoplasma mycoides* Cluster’ coincides with domestication of ruminants. PLoS One.

[7] Westberg J, Persson A, Holmberg A, Goesmann A, Lundeberg J, Johansson KE (2004). The genome sequence of *Mycoplasma mycoides* subsp. *mycoides* SC type strain PG1^T^, the causative agent of contagious bovine pleuropneumonia (CBPP). Genome Res.

[8] Wise KS, Calcutt MJ, Foecking MF, Madupu R, DeBoy RT, Roske K (2012). Complete genome sequences of *Mycoplasma leachii* strain PG50T and the pathogenic *Mycoplasma mycoides* subsp. *mycoides* small colony biotype strain Gladysdale. J Bacteriol.

[9] Orsini M, Krasteva I, Marcacci M, Ancora M, Ciammaruconi A, Gentile B, et al. Whole-Genome Sequencing of *Mycoplasma mycoides* subsp. *mycoides* Italian Strain 57/13, the Causative Agent of Contagious Bovine Pleuropneumonia. Genome Announc 2015;3(2). 10.1128/genomeA.00197-15 PMC4384145 25814605

[10] Bischof DF, Janis C, Vilei EM, Bertoni G, Frey J (2008). Cytotoxicity of *Mycoplasma mycoides* subsp. *mycoides* small colony type to bovine epithelial cells. Infect Immun.

[11] Bischof DF, Vilei EM, Frey J (2006). Genomic differences between type strain PG1 and field strains of *Mycoplasma mycoides* subsp. *mycoides* small-colony type. Genomics.

[12] Griffin RM (1969). Antigenic relationships among strains of *Mycoplasma mycoides* var. *mycoides*, *M. capri* and *M. laidlawii* revealed by complement-fixation tests. J Gen Microbiol.

[13] Jores J, Mariner JC, Naessens J (2013). Development of an improved vaccine for contagious bovine pleuropneumonia: an African perspective on challenges and proposed actions. Vet Res.

[14] Jores J, Nkando I, Sterner-Kock A, Haider W, Poole J, Unger H (2008). Assessment of *in vitro* interferon-gamma responses from peripheral blood mononuclear cells of cattle infected with *Mycoplasma mycoides* ssp. *mycoides* small colony type. Vet Immunol Immunopathol.

[15] Mulongo MM, Frey J, Smith K, Schnier C, Wesonga H, Naessens J (2013). Cattle immunized against the pathogenic L-alpha-glycerol-3-phosphate oxidase of *Mycoplasma mycoides* subs. *mycoides* fail to generate neutralizing antibodies and succumb to disease on challenge. Vaccine.

[16] Nkando I, Ndinda J, Kuria J, Naessens J, Mbithi F, Schnier C (2012). Efficacy of two vaccine formulations against contagious bovine pleuropneumonia (CBPP) in Kenyan indigenous cattle. Res Vet Sci.

[17] Sacchini F, Naessens J, Awino E, Heller M, Hlinak A, Haider W (2011). A minor role of CD4+ T lymphocytes in the control of a primary infection of cattle with *Mycoplasma mycoides* subsp. *mycoides*. Vet Res.

[18] Schieck E, Liljander A, Hamsten C, Gicheru N, Scacchia M, Sacchini F, et al. High antibody titres against predicted *Mycoplasma* surface proteins do not prevent sequestration in infected lung tissue in the course of experimental contagious bovine pleuropneumonia. Vet Microbiol. 2014;172(1–2):285–93. 10.1016/j.vetmic.2014.04.018 24880898

[19] Reynolds ES (1963). The use of lead citrate at high pH as an electron-opaque stain in electron microscopy. J Cell Biol.

[20] Hegermann J, Herrmann R, Mayer F (2002). Cytoskeletal elements in the bacterium *Mycoplasma pneumoniae*. Naturwissenschaften.

[21] Krause DC (1996). *Mycoplasma pneumoniae* cytadherence: unravelling the tie that binds. Mol Microbiol.

[22] Regula JT, Boguth G, Gorg A, Hegermann J, Mayer F, Frank R (2001). Defining the mycoplasma ‘cytoskeleton’: the protein composition of the Triton X-100 insoluble fraction of the bacterium *Mycoplasma pneumoniae* determined by 2-D gel electrophoresis and mass spectrometry. Microbiology.

[23] Seto S, Layh-Schmitt G, Kenri T, Miyata M (2001). Visualization of the attachment organelle and cytadherence proteins of *Mycoplasma pneumoniae* by immunofluorescence microscopy. J Bacteriol.

[24] Yaya A, Manso-Silvan L, Blanchard A, Thiaucourt F (2008). Genotyping of *Mycoplasma mycoides* subsp. *mycoides* SC by multilocus sequence analysis allows molecular epidemiology of contagious bovine pleuropneumonia. Vet Res.

[25] Jores J, Fischer A, Sirand-Pugnet P, Thomann A, Liebler-Tenorio EM, Schnee C (2013). *Mycoplasma feriruminatoris* sp. nov., a fast growing *Mycoplasma* species isolated from wild *Caprinae*. Syst Appl Microbiol.

[26] Galens K, Orvis J, Daugherty S, Creasy HH, Angiuoli S, White O (2011). The IGS standard operating procedure for automated prokaryotic annotation. Stand Genomic Sci.

[27] Field D, Garrity G, Gray T, Morrison N, Selengut J, Sterk P (2008). The minimum information about a genome sequence (MIGS) specification. Nat Biotechnol.

[28] Koren S, Schatz MC, Walenz BP, Martin J, Howard JT, Ganapathy G (2012). Hybrid error correction and de novo assembly of single-molecule sequencing reads. Nat Biotechnol.

[29] Miller JR, Delcher AL, Koren S, Venter E, Walenz BP, Brownley A (2008). Aggressive assembly of pyrosequencing reads with mates. Bioinformatics.

[30] Kurtz S, Phillippy A, Delcher AL, Smoot M, Shumway M, Antonescu C (2004). Versatile and open software for comparing large genomes. Genome Biol.

[31] Chin CS, Alexander DH, Marks P, Klammer AA, Drake J, Heiner C (2013). Nonhybrid, finished microbial genome assemblies from long-read SMRT sequencing data. Nat Methods.

[32] Hyatt D, Chen GL, Locascio PF, Land ML, Larimer FW, Hauser LJ (2010). Prodigal: prokaryotic gene recognition and translation initiation site identification. BMC Bioinformatics.

[33] Yu NY, Wagner JR, Laird MR, Melli G, Rey S, Lo R (2010). PSORTb 3.0: improved protein subcellular localization prediction with refined localization subcategories and predictive capabilities for all prokaryotes. Bioinformatics.

[34] Juncker AS, Willenbrock H, Von Heijne G, Brunak S, Nielsen H, Krogh A (2003). Prediction of lipoprotein signal peptides in Gram-negative bacteria. Protein Sci.

[35] Krogh A, Larsson B, von Heijne G, Sonnhammer EL (2001). Predicting transmembrane protein topology with a hidden Markov model: application to complete genomes. J Mol Biol.

[36] Sonnhammer EL, von Heijne G, Krogh A (1998). A hidden Markov model for predicting transmembrane helices in protein sequences. Proc Int Conf Intell Syst Mol Biol.

[37] Angiuoli SV, Matalka M, Gussman A, Galens K, Vangala M, Riley DR (2011). CloVR: a virtual machine for automated and portable sequence analysis from the desktop using cloud computing. BMC Bioinformatics.

[38] Riley DR, Angiuoli SV, Crabtree J, Dunning Hotopp JC, Tettelin H (2012). Using Sybil for interactive comparative genomics of microbes on the web. Bioinformatics.

[39] Woese CR, Kandler O, Wheelis ML (1990). Towards a natural system of organisms: proposal for the domains Archaea, Bacteria, and Eucarya. Proc Natl Acad Sci U S A.

[40] Schleifer K-H. Phylum XIII.Firmicutes. Paul De Vos, George M. Garrity, Dorothy Jones, Noel R. Krieg, Wolfgang Ludwig, Fred A. Rainey, Karl-Heinz Schleifer, Whitman WB, editors. Bergey's Manual of Systematic Bacteriology. New York: Springer; 2009;3

[41] Brown DR, May M, Bradbury JR, Johansson K-E. Phylum XVI. Tenericutes. Bergey's Manual of Systematic Bacteriology. Krieg NR, Ludwig W, Whitman W, Hedlund BP, Paster BJ, Staley JT, Ward N, Brown D, Parte A, editors. New York: Springer; 2010;4.

[42] Ludwig W, Euzéby J, Whitman WB. Road map of the phyla *Bacteroidetes*, *Spirochaetes*, *Tenericutes* (*Mollicutes*), *Acidobacteria*, *Fibrobacteres*, *Fusobacteria*, *Dictyoglomi*, *Gemmatimonadetes*, *Lentisphaerae*, *Verrucomicrobia*, *Chlamydiae*, and *Planctomycetes*. Krieg NR, Ludwig W, Whitman W, Hedlund BP, Paster BJ, Staley JT, Ward N, Brown D, Parte A, editors. New York: Springer; 2010

[43] Murray RGE. Bergey's Manual of Systematic Bacteriology. The Higher Taxa, or, a Place for Everything…?. Garrity G, Boone DR, Castenholz RW, editors. Baltimore: The William and Wilkins co.; 1984;1.

[44] Ed L (1984). Validation of the publication of new names and new combinations previously effectively published outside the IJSB. Int J Syst Bacteriol.

[45] Edward DG, Freundt EA (1973). Type strains of Species of the Order *Mycoplasmatales*, Including Designation of Neotypes for *Mycoplasma mycoides* subsp. *mycoides*, *Mycoplasma agalactiae* subsp. *agalactiae*, and *Mycoplasma arthritidis*. Int J Syst Bacteriol.

[46] Freundt EA (1955). The classification of the pleuropneumonia group of organisms (Borrelomycetales). Int Bull Bacteriological Nomenclature and Taxonomy.

[47] Ashburner M, Ball CA, Blake JA, Botstein D, Butler H, Cherry JM (2000). Gene ontology: tool for the unification of biology. The Gene Ontology Consortium. Nat Genet.

[48] Guindon S, Dufayard JF, Lefort V, Anisimova M, Hordijk W, Gascuel O. New algorithms and methods to estimate maximum-likelihood phylogenies: assessing the performance of PhyML 3.0. Syst Biol. 2010;59(3):307–21. 10.1093/sysbio/syq010 20525638

